# The Growth and Scope of Voluntary Hospitals in London

**Published:** 1898-01-08

**Authors:** 


### The Institutional Workshop.

### THE GROWTH AND SCOPE OF VOLUNTARY HOSPITALS IN LONDON.

(From a Correspondent.)

### XVII.—THE ANTISEPTIC SYSTEM OF TREATMENT.

Among the many influences which have conspired to bring the hospitals to their present state of efficiency one of the most important is the introduction of the antiseptic method of treating wounds, and the general recognition of the connection of fermentation, decomposition, suppuration, and septic processes with the presence and growth of micro-organisms capable of maintaining an independent life.

The first suggestion of the real source of septic mischief came to Lord Lister—then plain Mr. Lister—from reading a treatise of Pasteur's in 1857 on "Milk Fermentation," and his attention from that time began to be steadily directed towards the discovery of some counteracting agency to the deadly maladies which frustrated his surgical work in the Royal Glasgow Infirmary. "My patients," he writes, "suffered from the evils alluded to" (hospital gangrene, pyæmia, and erysipelas) "in a way that was sickening, and often heartrending, so as to make me feel it a questionable privilege to be connected with the institution." The severity of the scourge impelled him to action long before his theories were ripe or his methods definitely thought out. In 1864 he was struck with an account of the effect produced by carbolic acid on the sewage of Carlisle, and he began immediately to experiment with carbolic in his accident ward.

The situation of the new Surgical Hospital, built in 1860, and attached to the Glasgow Royal Infirmary, could hardly have been better selected had the main object of the architect been the testing of antiseptic theories. A few inches below the surface of the ground behind the two accident wards, with only the basement area, 4 ft. wide, between, was found the uppermost tier of a multitude of coffins dating from the cholera epidemic of 1849, in so imperfect a condition of decomposition that the clothes were still distinguishable. This was at one end of the building. The other end of the hospital was conterminous with the old cathedral churchyard, where pit burial prevailed, and it was computed that 5,000 bodies were lying in pits, holding 80 each, round the infirmary. And as if this were not enough, on a slight eminence above the churchyard lay an extensive cemetery, while the salubrity of the wards was further completed by the erection of the fever hospital at right angles to the surgical wing, and separated from it by only 8 ft. It remains to add that the infirmary contained 584 beds on an area of two acres, and was always full to overflowing, so that Lister had continual struggles to keep his wards from being crowded up with additional beds.

It was on this battle-field that the struggle with septic germ was fought out. The first experiment with carbolic acid were made by Lister, in March, 1865, in the Glasgow Royal Infirmary, on a case of compound fracture of the leg, and proved entirely unsuccessful. In August of the same year he made another trial—again a compound fracture of the leg, which he treated with lint dipped in carbolic acid, and this time he had the exquisite pleasure of seeing the patient's wounds heal quickly and well, in spite of the poisonous atmosphere of the wards. Another disappointing failure the following month completed the experiments for that year, since no further compound fractures occurred for eight months to lend an opportunity for observations. In 1866 his experiments were renewed, and after several conspicuous successes he became more confident, and before long he was able to save limbs under injuries which, previous to this discovery, would have compelled him to resort to amputation. By 1867 he was able to support his theory by the entire absence of pyæmia and gangrene from his accident ward on the ground floor, the adjoining accident ward of his fellow surgeon having meantime earned so deadly a character as to lead to its being closed and the horrible state of affairs underground, which we have already indicated, discovered.

All this time the antiseptic methods had been practised cautiously and tentatively, and had been discussed by Lister merely in his own immediate circle of friends and students. His first public announcement of the "new method of treating compound fractures, abscesses, &c," was made in the *Lancet*, March 16th, 1867, when he presented records of several cases in detail.

Many causes conspired, however, to prevent Lister's methods meeting with immediate acceptance. On the one hand, it was not every hospital in which the surroundings were so bad as they were at Glasgow. Erysipelas and pyæmia might occur, and indeed might prevail, now and again. But at most hospitals for considerable periods at a time surgeons met with a success which was not markedly inferior to what Lister was obtaining by his earlier methods. Again, the insistance laid by him upon aerial infection, and the way in which in the early days of antiseptic work failure was attributable to what appeared to be trifling lapses in the conduct of the method led many practical surgeons to look slightingly upon a system which seemed to demand a slavish following of a particular routine. If Lister's cases fail, they said, in consequence of a moment's exposure, how is it that ours succeed when they are exposed all the time? And no complete answer to this question was just then ready.

Gradually, however, the true principles of the antiseptic system began to be better understood, while it became clear that if they were borne in mind many methods were available. Antiseptics and antiseptic precautions of various kinds began to be usual at operations, antiseptic dressings largely replaced the old-fashioned lint, and then, as the wards became purer, as suppuration became less common, and as it became apparent that the mischief lay not so much in the air as in the actual transference of infection by instruments and bowls and the hands of the surgeons and the nurses, the aseptic system gradually came into being, the system by which antiseptics are applied to the unbroken skin, to the instruments, and the hands, but the wound is not touched, while the water and all the dressings are sterilised by heat.

Lister, fighting against insanitary conditions, which seemed universal and irremediable, had to trust to antiseptics, and his success was so great that the tendency was for a time to look upon the surroundings as unimportant. We read that Lister's ward was left (but this without his knowledge) for three years without the usual annual cleaning, and that on his at length remonstrating he was informed that the ward was so healthy as not to require cleaning. We read also of his venturing to introduce, on emergencies, extra beds and permitting two children to occupy the same cot, since he was able by the new treatment to avoid the ill-effects of an increase of numbers in the wards. There can be no doubt that, for a time, there was a danger of antiseptics leading to carelessness both among surgeons and hospital managers. That, however, soon passed over.

Far different has been the final effect on hospital work of the acceptance of the principles enunciated by Lister. From combating the septic germs disseminated by the air, the conflict gradually passed to guarding the air itself and the whole hospital from pollution.

Thus the exquisite cleanliness, brightness, and order which are now universally seen in the best hospitals, and are the keynote of successful hospital management, are a direct inheritance from Lord Lister. The first and the erroneous deduction drawn from his teaching was that dirt no longer mattered now that antiseptics had been discovered. A truer appreciation, however, of the principles which he had taught from the beginning, and which had never changed however much his methods varied, has now almost shown that antiseptics do not matter so long as there is no dirt.

